# Flavonoid Glycosides from *Ziziphus jujuba* var. *inermis* (Bunge) Rehder Seeds Inhibit α-Melanocyte-Stimulating Hormone-Mediated Melanogenesis

**DOI:** 10.3390/ijms22147701

**Published:** 2021-07-19

**Authors:** Ilandarage Menu Neelaka Molagoda, Kyoung-Tae Lee, Athapaththu Mudiyanselage Gihan Kavinda Athapaththu, Yung-Hyun Choi, Jaeyoung Hwang, Su-Jin Sim, Sanghyuck Kang, Gi-Young Kim

**Affiliations:** 1 Department of Marine Life Science, Jeju National University, Jeju 63243, Republic of Korea; neelakagm2012@gmail.com (I.M.N.M.); gihankavinda@yahoo.com (A.M.G.K.A.); 2 Research Institute for Basic Sciences, Jeju National University, Jeju 63243, Republic of Korea; 3 Forest Biomaterials Research Center, National Institute of Forest Science, Jinju 52817, Republic of Korea; leekt99@korea.kr (K.-T.L.); sujin0606@korea.kr (S.-J.S.); 4 Department of Biochemistry, College of Oriental Medicine, Dong-Eui University, Busan 47227, Republic of Korea; choiyh@deu.ac.kr; 5 Department of Chemistry, Gyeongsang National University, Jinju 52828, Republic of Korea; jaeyoung@gnu.ac.kr; 6 Korea Beauty Industry Development Institute, Jeju 63309, Republic of Korea; ksanggh@kbidi.or.kr

**Keywords:** *Ziziphus jujuba* var. *inermis* (Bunge) Rehder, flavonoid glycoside

## Abstract

*Ziziphus jujuba* extracts possess a broad spectrum of biological activities, such as antioxidant and anticancer activities in melanoma cancers. Nevertheless, the compounds contain high antioxidant capacities and anticancer activities in melanoma cells, shown to be effective in hyperpigmentation disorders, but whether flavonoid glycosides from *Z*. *jujuba* regulate anti-melanogenesis remains unclear. In this study, we evaluated the anti-melanogenic activity of five flavonoid glycosides from *Z. jujuba* var. *inermis* (Bunge) Rehder seeds, including jujuboside A (JUA), jujuboside B (JUB), epiceanothic acid (EPA), betulin (BTL), and 6’’’-feruloylspinosin (FRS), in B16F10 melanoma cells and zebrafish larvae. According to our results, JUB, EPA, and FRS potently inhibited α-melanocyte-stimulating hormone (α-MSH)-induced melanogenesis and prevented hyperpigmentation in zebrafish larvae. In particular, under α-MSH-stimulated conditions, FRS most significantly inhibited α-MSH-induced intracellular and extracellular melanin content in B16F10 melanoma cells. Additionally, JUB, EPS, and FRS remarkably downregulated melanogenesis in α-MSH-treated zebrafish larvae, with no significant change in heart rate. Neither JUA nor BTA were effective in downregulating melanogenesis in B16F10 melanoma cells and zebrafish larvae. Furthermore, JUB, EPA, and FRS directly inhibited in vitro mushroom tyrosinase enzyme activity. JUB, EPA, and FRS also downregulated cyclic adenosine monophosphate (cAMP) levels and the phosphorylation of cAMP-response element-binding protein (CREB), and subsequent microphthalmia transcription factor (MITF) and tyrosinase expression. In conclusion, this study demonstrated that JUB, EPA, and FRS isolated from *Z. jujuba* var. *inermis* (Bunge) Rehder seeds exhibit potent anti-melanogenic properties by inhibition of the cAMP-CERB-MITF axis and consequent tyrosinase activity.

## 1. Introduction

Skin pigmentation is naturally regulated by crosstalk between melanin-producing melanocytes and melanin-receiving keratinocytes [1]. Synthesized melanin protects the skin from ultraviolet radiation and free radicals [2]. Nevertheless, the excessive production of melanin leads to undesirable hyperpigmentation-related dermatological disorders, such as melisma, freckles, lentigines, and age spots [3,4]. Therefore, many scientists have sought natural compounds that prevent melanogenesis and that facilitate the resolution of hyperpigmentation.

Several cell signaling pathways are responsible for melanin synthesis. Among them, the α-melanocyte-stimulating hormone (α-MSH)-mediated upregulation of microphthalmia transcription factor (MITF) is considered a key route [5]. The binding of α-MSH to the melanocortin-1 receptor (MC1R) promotes cyclic adenosine monophosphate (cAMP) formation and phosphorylates cAMP-response element-binding protein (CREB) through protein kinase A (PKA), and consequently transactivates MITF [6]. Activated MITF subsequently enhances the transcription of melanogenesis-related proteins, such as tyrosinase, tyrosinase-related protein-1 (TRP-1), and TRP-2 [7]. The initial step of melanin synthesis involves the hydroxylation of L-tyrosine to dihydroxyphenylalanine (DOPA), and subsequent oxidation of DOPA to DOPA-quinone by tyrosinase, indicating that tyrosinase is a key rate-limiting enzyme in melanogenesis [8]. Therefore, the inhibition of tyrosinase might be an effective way to prevent hyperpigmentation-related disorders.

*Ziziphus jujuba* has been used as an effective sedative in traditional herbal medicine and contains abundant nutrients such as minerals and vitamins, triterpenoid acids, polysaccharides, and polyphenols [9,10]. To date, many flavonoids from the leaves, fruits, and seeds of *Z. jujuba* Mill. have been isolated: these induce anti-inflammatory, anticancer, antidiabetic, and neuroprotective effects [11,12,13]. Recently, Moon et al. [14] also demonstrated that spinosin isolated from *Z. jujuba* Mill. possesses anti-melanogenic properties in B16F10 melanoma cells and human skin models by inhibiting tyrosinase activity. However, whether other flavonoids from *Z. jujuba* inhibit melanogenesis remains unclear. Therefore, we evaluated the anti-melanogenic properties of five flavonoid glycosides: jujuboside A (JUA), jujuboside B (JUB), epiceanothic acid (EPA), betulin (BTL), and 6’’’-feruloylspinosin (FRS) from *Ziziphus jujuba* var. *inermis* (Bunge) Rehder seeds. In this study, we found that JUB, EPA, and FRS exhibited potent anti-melanogenic activity in both B16F10 melanoma cells and zebrafish larvae by inhibiting tyrosinase activity and downregulating the melanin-producing cell signaling pathway.

## 2. Results

### 2.1. No Cytotoxic Effects of Flavonoid Glycosides Were Presented in B16F10 Melanoma Cells

**Figure 1:**
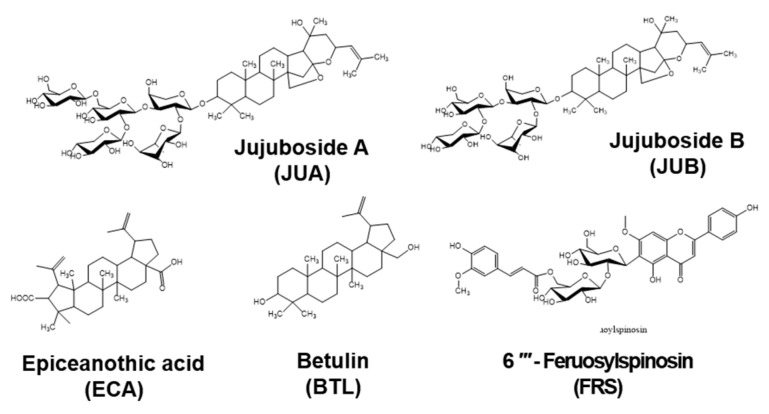
Chemical structure of flavonoid glycosides isolated from *Z. jujuba* var. *inermis* (Bunge) Rehder seeds.

**Figure 2:**
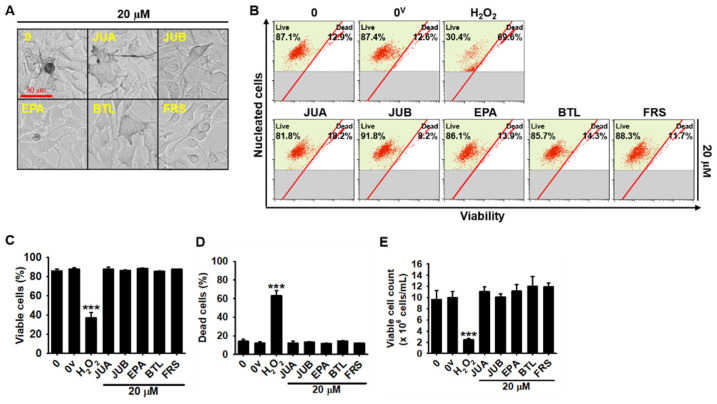
Flavonoid glycosides from *Z. jujuba* var. *inermis* (Bunge) Rehder seeds present no cytotoxicity in B16F10 melanoma cells. B16F10 melanoma cells (5 × 10^4^ cells/mL) were treated with 20 µM of jujuboside A (JUA), jujuboside B (JUB), epiceanothic acid (EPA), betulin (BTL), and 6′′′-feruloylspinosin (FRS) for 96 h. (**A**) Images of the cells were captured using a phase contrast microscope (×20). Scale bar = 50 µm. (**B**) Cell viability was analyzed by a Muse Cell Count and Viability Assay Kit. (**C**) Viable cell (%) and (**D**) dead cell (%) population, and (**E**) viable cell count (×10^6^ cells/mL) are shown. Hydrogen peroxide (H_2_O_2_, 100 µM) was used as a cellular death-inducing positive control. All data are presented as a standard error of the median from three independent experiments (*** *p* < 0.001 vs. untreated cells). 0, untreatment; 0^v^, vehicle control (0.01% DMSO).

The chemical structures of JUA, JUB, EPA, BTL, and FRS are shown in Figure 1. To evaluate the cytotoxic effects of JUA, JUB, EPA, BTL, and FRS, B16F10 melanoma cells were treated with 20 µM of flavonoid glycosides for 96 h, and cytotoxicity was evaluated based on morphological observations and flow cytometric analysis. As shown in Figure 2A, no cytotoxic hallmarks such as dead cells, apoptotic bodies, floating and round-shaped cells, or cell debris were observed in the presence of all flavonoid glycosides tested in this study. In a parallel experiment, we evaluated cytotoxicity in depth using flow cytometric analysis after 96 h (Figure 2B). Consistent with the previous morphological observations, the treatment with each flavonoid glycoside had no effect on the viable cell population (87.9 ± 2.0%, 86.6 ± 0.3%, 88.5 ± 0.5%, 85.8 ± 0.4%, and 87.9 ± 0.3% at JUA, JUB, EPA, BTL, and FRS) compared with that in untreated cells (86.0 ± 2.2%, Figure 2C). Furthermore, dead cell population (12.2 ± 2.0%, 13.4 ± 0.3%, 11.6 ± 0.5%, 14.2 ± 0.5%, and 12.1 ± 0.3% at JUA, JUB, EPA, BTL, and FRS, respectively, Figure 2D) and viable cell counts ((11.1 ± 0.8, 10.1 ± 0.6, 11.1 ± 1.2, 12.00 ± 1.8, and 11.9 ± 0.7) × 10^6^ cells/mL at JUA, JUB, EPA, BTL, and FRS, respectively, Figure 2E) were also not changed compared with those in untreated cells (14.0 ± 2.2% dead cell population and (9.7 ± 1.6) × 10^6^ cells/mL viable cell counts, respectively). However, hydrogen peroxide (H_2_O_2_) resulted in a 37.9 ± 1.4% viable cell population, 62.9 ± 5.7% dead cell population, and (2.4 ± 0.3) × 10^6^ cells/mL viable cell count. These data indicated that 20 µM of JUA, JUB, EPA, BTL, and FRS had no effect on the viability of B16F10 melanoma cells.

### 2.2. JUB, EPA, and FRS Inhibit α-MSH-Induced Melanogenesis in B16F10 Melanoma Cells

**Figure 3:**
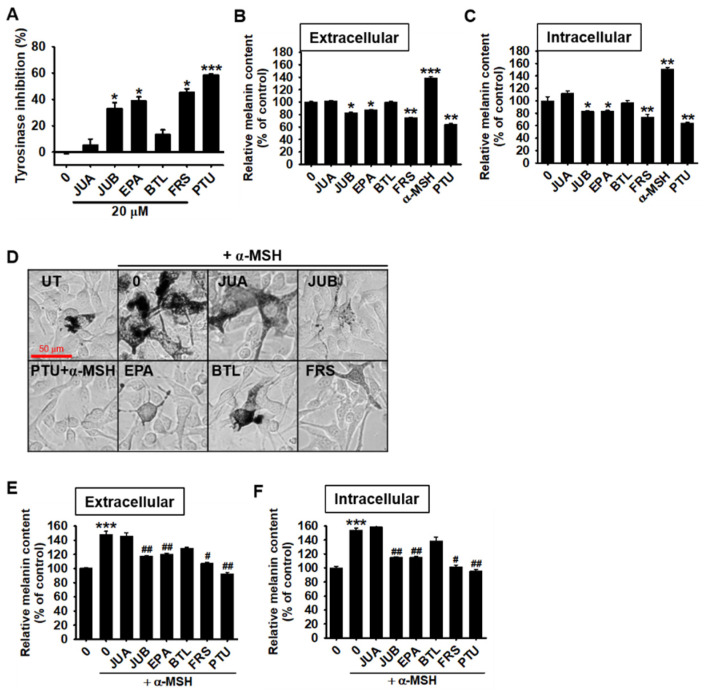
JUB, BTL, and FRS from *Z. jujuba* var. *inermis* (Bunge) Rehder seeds inhibit in vitro tyrosinase activity and melanin production in B16F10 melanoma cells. (**A**) In vitro mushroom tyrosinase enzyme activity in the presence of 20 µM of jujuboside A (JUA), jujuboside B (JUB), epiceanothic acid (EPA), betulin (BTL), and 6′′′-feruloylspinosin (FRS) was determined by oxidation of L-tyrosinase as a substrate. Phenylthiourea (PTU, 200 nM) was used as a positive control. B16F10 murine melanoma cells (5 × 10^4^ cells/mL) were treated with 20 µM of JUA, JUB, EPA, BTL, and FRS for 96 h in the (**B**,**C**) absence and (**D**–**F**) presence of 500 ng/mL of α-melanocyte-stimulating hormone (α-MSH). Intracellular and extracellular melanin was quantified. PTU was used as a negative control. (**D**) Images of the cells were taken with a phase contrast microscope (×20). Scale bar = 50 µm. All data are presented as standard errors of the median from three independent experiments (*** *p* < 0.001, ** *p* < 0.01, and * *p* < 0.05 vs. untreated cells; ^##^ *p* < 0.01 and ^#^ *p* < 0.05 vs. α-MSH-treated cells).

We investigated in vivo mushroom tyrosinase activity to identify the anti-melanogenic properties of each flavonoid glycoside. As shown in Figure 2A, JUB, EPA, and FRS markedly increased the inhibitory rate of mushroom tyrosinase activity to 33.9 ± 4.5%, 38.4 ± 3.1%, and 45.7 ± 2.7%, respectively, compared with those of untreated cells (Figure 3A). However, JUA (7.4 ± 5.3%) and BTL (11.2 ± 5.1%) slightly inhibited mushroom tyrosinase enzyme activity, but this inhibition was not statistically significant. Phenylthiourea (PTU, 200 nM) was used as a positive control and intensively inhibited in vitro mushroom tyrosinase activity to 58.4 ± 1.0%. The above data showed that certain flavonoid glycosides from *Z. jujuba* var. *inermis* (Bunge) Rehder seeds, including JUB, EPA, and FRS, directly inhibited tyrosinase activity, whereas JUA and BTL did not. Additionally, JUB, EPA, and FRS decreased the spontaneous production (in the absence of α-MSH) of extracellular (Figure 3B) and intracellular (Figure 3C) melanin in B16F10 melanoma cells. In detail, JUB, EPA, and FRS downregulated extracellular melanin content to 82.6 ± 1.7%, 87.5 ± 0.8%, and 74.8 ± 0.8%, and intracellular melanin content to 83.0 ± 1.1%, 83.2 ± 1.7%, and 73.9 ± 4.2%, respectively, compared with untreated cells. PTU also significantly downregulated extracellular and intracellular and melanin content to 63.6 ± 2.6% and 64.2 ± 1.4%, respectively. B16F10 melanoma cells were treated with 20 µM of flavonoid glycosides in the presence and absence of α-MSH for 96 h. As expected, JUB, EPA, and FRS inhibited α-MSH-induced pigmentation in B16F10 melanoma cells, whereas the treatment with JUA and BTL did not have any apparent effects (Figure 3D). Consistent with the above data, under α-MSH stimulation, JUB, EPA, and FRS inhibited the extracellular melanin content from 147.7 ± 5.2% to 117.4 ± 0.5%, 119.9 ± 2.0%, and 106.8 ± 1.9%, respectively (Figure 3E), and the intracellular melanin content from 153.5 ± 3.3% to 114.7 ± 1.5%, 113.8 ± 2.1%, and 101.4 ± 2.9%, respectively (Figure 3F). In particular, the inhibitory levels of FRS were comparable to those of PTU (92.3 ± 2.4% and 95.3 ± 30.2% intracellular and extracellular melanin content in the presence of α-MSH, respectively). No significant inhibitory effect was observed in JUA- and BTL-treated cells in the presence or absence of α-MSH. These data indicated that among the flavonoid glycosides tested in this study, only JUB, EPA, and FRS showed potent anti-melanogenic properties in B16F10 melanoma cells.

### 2.3. JUB, EPA, and FRS Prevent α-MSH-Induced Melanogenesis in Zebrafish Larvae

**Figure 4:**
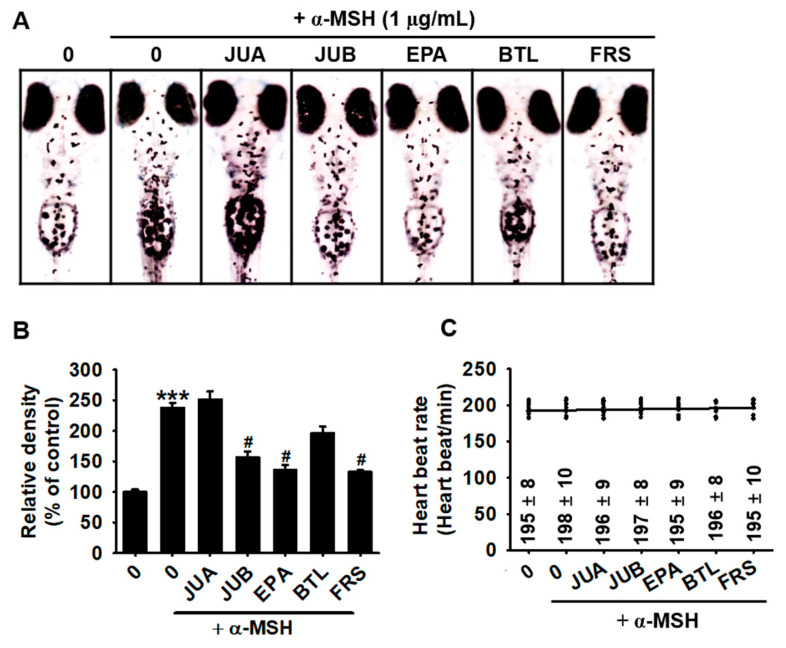
JUB, BTL, and FRS from *Z. jujuba* var. *inermis* (Bunge) Rehder seeds inhibit α-MSH-induced pigmentation in zebrafish larvae. (**A**) Zebrafish larvae at 3 dpf (*n* = 20 in each group) were treated with 20 µM of jujuboside A (JUA), jujuboside B (JUB), epiceanothic acid (EPA), betulin (BTL), and 6’’’-feruloylspinosin (FRS) for 72 h in the presence and absence of 1 µg/mL of α-MSH. (A) Images of zebrafish larvae were captured using a stereomicroscope (×4). (**B**) Relative melanocyte densities of zebrafish larvae were calculated using ImageJ software. (**C**) Heart beats were manually counted and expressed as heart beats/min. All data are presented as a standard error of the median (*** *p <* 0.001 vs. untreated zebrafish larvae; ^#^ *p* < 0.05 vs. α-MSH treated zebrafish larvae).

Next, we evaluated the anti-melanogenic properties of flavonoid glycosides in zebrafish larvae. Zebrafish larvae at 3 days post-fertilization (dpf) were treated with 20 µM of JUA, JUB, EPA, BTL, and FRS in the presence and absence of α-MSH for 72 h. Three of these compounds (JUB, EPA, and FRS) significantly downregulated α-MSH-induced pigmentation in the developing zebrafish larvae (Figure 4A). Compared with untreated zebrafish larvae, α-MSH-induced zebrafish pigmentation up to 237.7 ± 7.8%, whereas treatment with JUB, EPA, and FRS significantly inhibited α-MSH-induced pigmentation to 156.3 ± 10.0%, 136.2 ± 8.1%, and 133.1 ± 3.1%, respectively (Figure 4B). As expected, JUA and BTL had no significant anti-melanogenic effects in α-MSH-treated zebrafish larvae. In addition, heart rate measurements showed that none of the tested compounds were cardiotoxic in zebrafish larvae at a concentration of 20 µM compared with that in untreated larvae (Figure 4C). These results showed the anti-melanogenic properties of JUB, EPA, and FRS in vivo, but not JUA and BTL.

### 2.4. Anti-Melanogenic Properties of JUB, EPA, and FRS Are Related to Inhibition of MITF and Tyrosinase Expression

**Figure 5:**
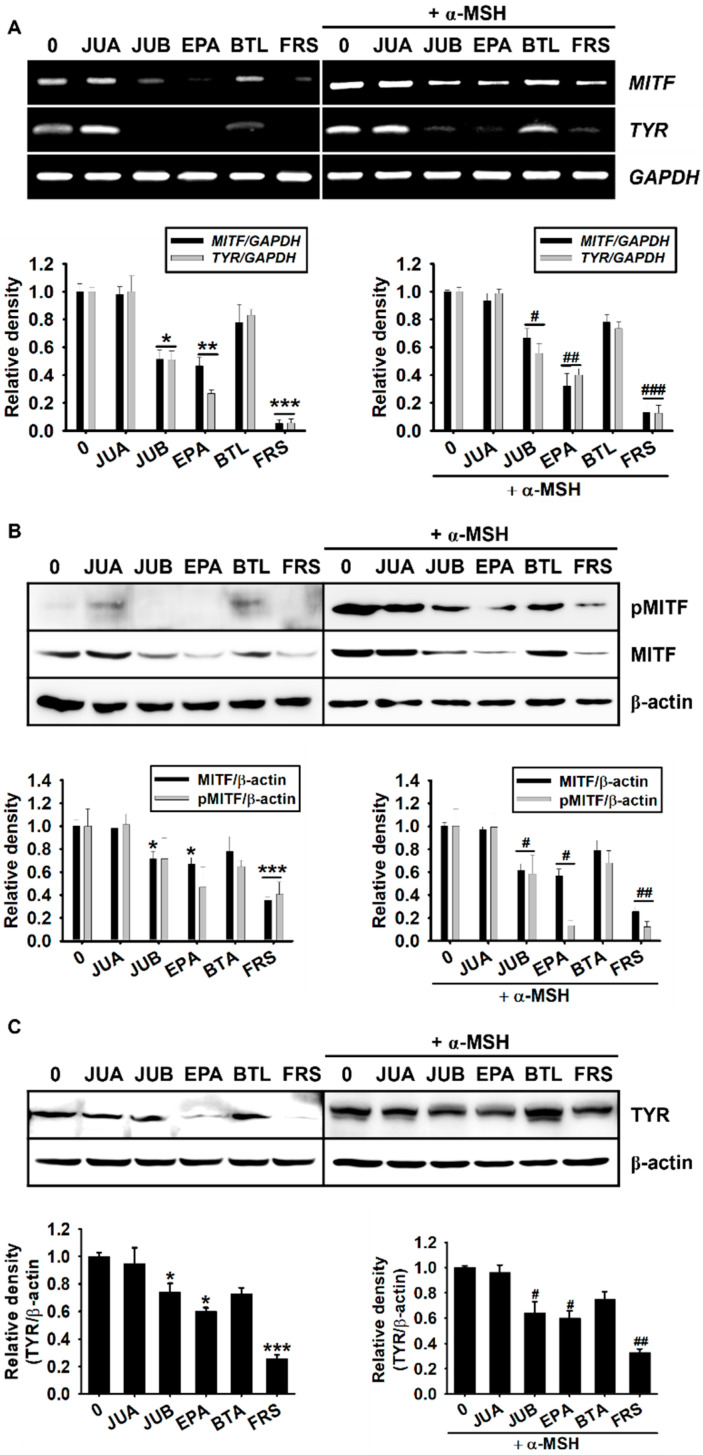
JUB, BTL, and FRS from *Z. jujuba* var. *inermis* (Bunge) Rehder seed downregulated MITF and tyrosinase (TYR) expression. B16F10 melanoma cells (5 × 10^4^ cells/mL) were treated with 20 µM of jujuboside A (JUA), jujuboside B (JUB), epiceanothic acid (EPA), betulin (BTL), and 6’’’-feruloylspinosin (FRS) in the absence (*left*) and presence (*right*) of 500 ng/mL of α-melanocyte-stimulating hormone (α-MSH). (**A**) Reverse transcription-polymerase chain reaction (RT-PCR) was performed at 48 h to evaluate the gene expression of *microphthalmia-associated transcription factor* (*MITF*) and *tyrosinase* (*TYR*). *Glyceraldehyde 3-phosphate dehydrogenase* (*GAPDH*) was used as a loading control. (**B**,**C**) Western blotting was performed at 96 h to evaluate the protein expression of (**B**) pMITF and MITF and (**C**) TYR. β-Actin was used as the internal control. Relative densities were calculated from ImageJ software and intensity levels were normalized to the levels of (**A**) *GAPDH* and (**B**,**C**) β-actin. All data are presented as standard errors of the median from three independent experiments (*** *p <* 0.001, ** *p <* 0.01, and * *p* < 0.05 vs. untreated cells; ^###^ *p <* 0.001, *^##^ p* < 0.01, and ^#^ *p* < 0.05 vs. α-MSH-treated cells).

As MITF is considered a key transcription factor involved in melanogenesis by transactivating tyrosinase expression [5,6], we evaluated the effects of flavonoid glycosides on MITF and tyrosinase expression at both the transcriptional and translational levels. According to RT-PCR data, JUB, EPA, and FRS inhibited *MITF* and *tyrosinase* expression 48 h after treatment, both in the absence (Figure 5A, *left*) and presence (Figure 5A, *right*) of α-MSH. Consistent with RT-PCR data, JUB, EPA, and FRS markedly inhibited the total MITF-phosphorylated MITF protein expression 96 h after treatment, both in the absence (Figure 5B, *left*) and presence (Figure 5B, *right*) of α-MSH. As we expected, JUB, EPA, and FRS markedly inhibited the tyrosinase protein expression both in the absence (Figure 5C, *left*) and presence (Figure 5C, *right*) of α-MSH. However, no significant decrease was observed in JUA- or BTL-treated cells. Taken together, these results indicated that JUB, EPA, and FRS possess anti-melanogenic properties by inhibiting MITF and tyrosinase expression.

### 2.5. JUB, EPA, and FRS Inhibit cAMP-Mediated CREB Phosphorylation

**Figure 6:**
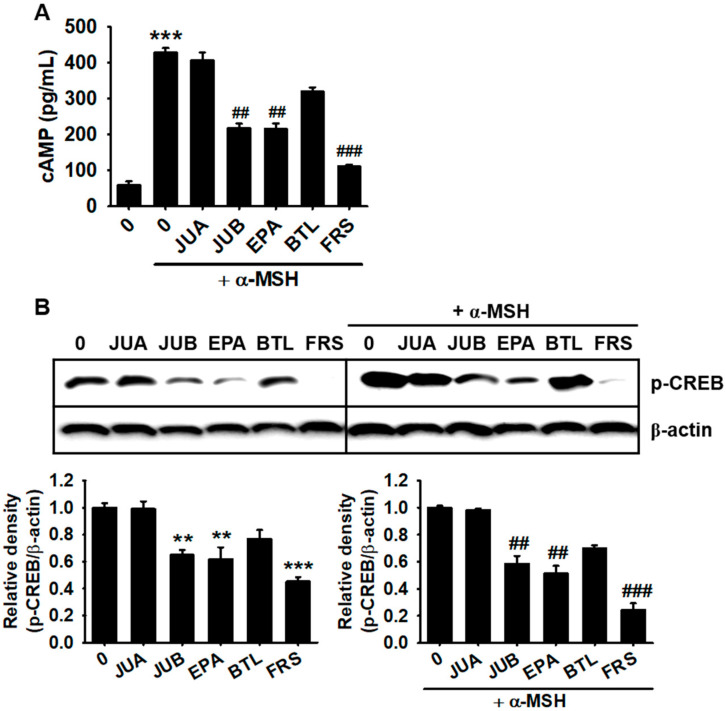
JUB, BTL, and FRS from *Z. jujuba* var. *inermis* (Bunge) Rehder seed inhibit cAMP levels and subsequent CREB phosphorylation. B16F10 melanoma cells (5 × 10^4^ cells/mL) were treated with 20 µM of jujuboside A (JUA), jujuboside B (JUB), epiceanothic acid (EPA), betulin (BTL), and 6’’’-feruloylspinosin (FRS) in the presence and absence of 500 ng/mL of α-melanocyte-stimulating hormone (α-MSH). (**A**) Cyclic adenosine monophosphate (cAMP) levels were quantified at 15 min by enzyme-linked immunosorbent assay. (**B**) Western blotting was performed at 96 h to evaluate the expression of phosphorylated cAMP response element-binding protein (CREB). β-Actin was used as the internal control. Relative densities were calculated using ImageJ software and intensity levels were normalized to those of β-actin. All data are presented as standard errors of the median from three independent experiments (*** *p <* 0.001 and ** *p* < 0.01 vs. untreated cells; *^###^ p* < 0.001 and *^##^ p* < 0.01 vs. α-MSH-treated cells).

As α-MSH-mediated melanogenic signals are mainly transduced through the cAMP-CREB-MITF axis [15,16], we evaluated the effects of flavonoid glycosides on α-MSH-induced cAMP levels and subsequent CREB phosphorylation. According to cAMP enzyme-linked immunosorbent assay (ELISA) results, 15 min after α-MSH treatment, cAMP levels were significantly increased to 429.1 ± 12.2 pg/mL (Figure 6A). As expected, JUB, EPA, and FRS potently inhibited α-MSH-induced cAMP levels to 217.9 ± 13.3, 215.9 ± 15.5, and 111.2 ± 5.7 pg/mL, respectively (Figure 6A), whereas neither JUA (406.7 ± 22.3 pg/mL) nor BTL (321.0 ± 10.7 pg/mL) had significant effects. As depicted in Figure 6B, JUB, EPA, and FRS were effective in downregulating CREB phosphorylation regardless of the absence (*left*) and presence (*right*) of α-MSH, whereas JUA and BTA were not able to inhibit CREB phosphorylation. These results suggested that JUB, EPA, and FRS inhibit cAMP and subsequent CREB phosphorylation.

## 3. Discussion

The use of natural compounds to treat or prevent hyperpigmentation-related skin problems has become popular recently owing to the adverse side effects and cytotoxicity of currently available anti-melanogenic agents on skin cells [17]. Fruits and seeds of *Z. jujuba* Mill. var. *spinosa* (Bunge) possess extensive sedative and hypnotic effects [18] and are therefore considered an important raw material in the food and pharmaceutical industries [19]. Recent investigations of Guo et al. demonstrated that 100 g of *Ziziphus jujuba* var. *spinosa* contains 73.11 ± 33.90, 79.21 ± 24.17, 17.57 ± 5.35, 59.15 ± 22.25, and 30.81 ± 11.65 mg of spinosin, JUA, JUB, BTL, and FRS, respectively [20]. Jujube seed extracts analyzed by HPLC in conjunction with DAD and MS detection revealed that the seeds are rich with flavonoids and free amino acids, while the isolated components were associated with strong anti-oxidative properties and proven to be beneficial in food processing [21]. In detail, JUB, one of the most prevalent flavonoids in Zizyphi Spinosi Semen, suppresses tumor formation, platelet aggregation, and vascular tension [22,23,24]. EPA is a pentacyclic triterpene possessing strong anti-HIV properties [25,26]. FRS protects the heart against acute myocardial ischemia and reperfusion injury in a rat model and alleviates beta-amyloid-induced toxicity in *Caenorhabditis elegans* (GMC101) and PC12 cells [27]. In addition, JUA and BTL also ubiquitously exist in Zizyphi Spinosi Semen [28,29] and show potent sedative and hypnotic effects [30]. Recently, eight triterpenoids have been isolated from *Z. jujuba* var. *inermis* (Bunge) Rehder, which induces apoptotic death in human cancer cells by activating mitochondrial reactive oxygen species production [31]. Tran et al. [11] found three new flavonoids from *Z. jujuba* var. *inermis* (Bunge) Rehder, which exhibit anti-inflammatory and cytotoxic activities. Besides, cAMP was found to be abundant in Jujube seed [32], and Moon et al. [14] determined that spinosin from the seed of *Z*. *jujuba* prevents skin pigmentation by inhibiting tyrosinase. In addition, whether the other bioactive compounds such as flavonoids isolated from *Z. jujuba* var. *inermis* (Bunge) Rehder seeds are involved in melanogenesis is poorly understood. In this study, five flavonoid glycosides purified from *Z. jujuba* var. *inermis* (Bunge) Rehder seeds were supplied by the National Institute of Forest Science (Jinju, Geongsangnam-do, Republic of Korea), and their anti-melanogenic effects in α-MSH-stimulated B16F10 melanoma cells and zebrafish larvae were investigated. According to our results, among the flavonoid glycosides tested in this study, only JUB, EPA, and FRS effectively inhibited α-MSH-induced melanogenesis in both B16F10 melanoma cells and zebrafish larvae by inhibiting the cAMP-CREB-MITF-tyrosinase axis.

cAMP is considered to be a key signaling molecule that regulates pigmentation by activating PKA-mediated CREB phosphorylation through the binding of α-MSH to MC1R [15]. Therefore, natural compounds that target the cAMP-PKA-CREB axis act as powerful anti-melanogenic agents [5,33]. Additionally, CREB transactivates MITF expression, which promotes the transcription of tyrosinase, leading to increased melanin production [16]. Previously, Chen et al. [34] reported that knockout of the *MITF-M* gene results in albinism in mice, confirming the significance of MITF as a master transcription factor for melanin production. Additionally, small interfering RNA-mediated *MITF* silencing in melanoma cells significantly reduced melanin content by inhibiting tyrosinase, TRP-1, and MC1R [35], indicating that inhibition of the transcriptional activation of MITF is also a promising strategy to treat hypermelanogenic disorders. In this study, JUB, EPA, and FRS were identified as potent inhibitors of cAMP, CREB, and MITF expression, suggesting that the cAMP-PKA-CREB-MITF axis may be responsible for the anti-melanogenic properties of JUB, EPA, and FRS. Particularly, tyrosinase is considered to be the key rate-limiting enzyme in the melanogenic pathway [8]; hence, most of the currently available anti-melanogenic agents such as hydroquinone, arbutin, and aloesin target enzyme activity in a reversible or irreversible manner [36]. In this study, in addition to the inhibition of the cAMP-PKA-CREB-MITF axis, direct inhibition of mushroom tyrosinase enzyme activity also enabled JUB, EPA, and FRS to act as potent anti-melanogenic agents. We also attempted to perform molecular docking simulations between flavonoid glycosides and tyrosinase but failed to predict the 3D structure of flavonoid glycosides because of the structural complexity. Nevertheless, according to the in vitro mushroom tyrosinase enzyme activity, we believe that JUB, EPA, and FRS may directly bind to tyrosinase. On the other hand, JUA and BTL did not directly inhibit mushroom tyrosinase activity in vitro or melanogenesis in B16F10 melanoma cells and zebrafish larvae. Whether the lack of any significant effect on melanogenesis by JUA and BTL is due to the structural difference or concentrations used in this study remains unknown. Therefore, higher concentrations than those employed in this study are needed for further evaluation of the JUA and BTL involvement in melanogenesis.

## 4. Materials and Methods

### 4.1. Reagents and Antibodies

Flavonoid glycosides isolated from *Z. jujuba* var. *inermis* (Bunge) Rehder seeds, including JUA, JUB, EPA, BTL, and FRS (Figure 1), were provided from the National Institute of Forest Science (Jinju, Geongsangnam-do, Republic of Korea). Mushroom tyrosinase, L-tyrosine α-MSH, and PTU were purchased from Sigma-Aldrich Chemical Co. (St. Louis, MO, USA). Dulbecco’s modified Eagle’s medium (DMEM), fetal bovine serum (FBS), and a penicillin-streptomycin (50×) antibiotic mixture were purchased from WELGENE (Gyeongsan, Gyeongsangbuk-do, Republic of Korea). Antibodies against MITF (sc-71588, 60 kDa), tyrosinase (sc-20035, 84 kDa), phospho (p)-CREB (sc-81486, 43 kDa), β-actin (sc-69879, 43 kDa), and peroxidase-labeled anti-mouse immunoglobulins (sc-516102) were purchased from Santa Cruz Biotechnology (Santa Cruz, CA, USA).

### 4.2. Cell Culture

B16F10 melanoma cells were obtained from American Type Culture Collection (Manassas, VA, USA). The cells were maintained at 37 °C in a 5% CO_2_ humidified incubator in DMEM supplemented with 10% heat-inactivated FBS and antibiotic mixture.

### 4.3. Cell Viability and Morphology

Viable cell and dead cell population, and viable cell count were measured using flow cytometry (Luminex, Austin TX, USA). Briefly, B16F10 melanoma cells were seeded at a density of 5 × 10^4^ cells/mL in 6-well plates and incubated with 20 µM of flavonoid glycosides for 96 h. Hydrogen peroxide (H_2_O_2_, 100 µM) was used as the cell death-inducing control. After harvesting, the cells were incubated with a Muse Cell Count and Viability Kit (Luminex) for 5 min. Viable cell and dead cell population, and viable cell count were analyzed by a Muse Cell Cycler (Luminex). In a parallel experiment, cellular images were captured using a phase-contrast microscope (MACROTECH, Goyang, Gyeonggi-do, Republic of Korea).

### 4.4. In Vitro Mushroom Tyrosinase Activity

In vitro mushroom tyrosinase activity was determined by the methods of Duckworth and Coleman with some modifications [37]. Briefly, 130 µL of 100 mM of phosphate buffer (pH 6.8), 20 µL of flavonoid glycosides, and 30 µL of 1.5 mM of L-tyrosine were mixed. Finally, 20 µL of 210 Units/mL of mushroom tyrosinase was mixed and then incubated for 10 min at 37 °C. Absorbance was measured at 490 nm using a microplate spectrophotometer (BioTek Instruments Inc., Winooski, VT, USA). PTU (250 nM) was used as a negative control. Mushroom tyrosinase inhibition (%) was calculated using:

Inhibition (%) = [1 − (A/B)] × 100(1)

where A is the absorbance of the test compound and B is the absorbance of the blank.

### 4.5. Quantification of Extracellular and Intracellular Melanin Content

B16F10 melanoma cells were seeded at a density of 5 × 10^4^ cells/mL in 6-well plates and treated with 20 µM of flavonoid glycosides for 96 h in the presence or absence of 500 ng/mL of α-MSH. Then, culture media and cell pellets were separately collected to quantify extracellular and intracellular melanin contents, respectively [29]. The absorbance of culture media was measured at 405 nm for extracellular melanin content. The cell pellets were washed with ice-cold PBS and were dissolved in 400 µL of 1 M of NaOH containing 10% dimethyl sulfoxide and heated at 90 °C for 60 min. Then, the cells were centrifuged with a table-top centrifuge at 1000 RPM for 3 min at room temperature to remove any remaining debris. Protein was quantified by Bio-Rad Protein Assay Reagents (Bio-Rad, Hercules, CA, USA). The total volume was adjusted to 200 µL and the absorbance of equal amounts of proteins was measured at 405 nm for intracellular melanin content.

### 4.6. Reverse Transcription-Polymerase Chain Reaction (RT-PCR)

B16F10 melanoma cells were cultured at a density of 5 × 10^4^ cells/mL in 6-well plates and treated with 20 µM of flavonoid glycosides in the presence and absence of 500 ng/mL of α-MSH for 48 h. Total RNA was extracted using TRIzol Reagent (Life Technologies, Carlsband, CA, USA) according to the manufacturer’s instruction. RNA was reverse-transcribed using MMLV reverse transcriptase (Bioneer, Daejeon, Republic of Korea). The cDNA was amplified using a EzWay PCR Ready Mix (KOMA BIOTECH, Seoul, Republic of Korea) with specific primers (Table 1) [38].

### 4.7. Protein Extraction and Western Blotting

The cells were lysed at 96 h with a RIPA Lysis Buffer (iNtRON Biotechnology, Sungnam, Gyeonggi-do, Republic of Korea) with protease inhibitors. Protein was quantified by Bio-Rad Protein Assay Reagents (Bio-Rad) and equal amounts of proteins were separated in sodium dodecyl sulfate-polyacrylamide gel, transferred onto a polyvinylidene fluoride membrane (Amersham, Arlington Heights, IL, USA), and then immunoblotted with the indicated primary (200 μg/mL, 1:1000 dilution) and secondary antibodies (400 μg/mL, 1:3000 dilution). Finally, chemiluminescence was observed using an enhanced chemiluminescence detection system (Amersham) and images were captured using ImageQuant LAS 500 (GE Healthcare Bio-Sciences AB, Uppsala, Sweden). The expression values were normalized relative to the intensity level of β-actin.

### 4.8. Measurement of cAMP

B16F10 melanoma cells were seeded at a density of 5 × 10^4^ cells/mL in 6-well plates and treated with 20 µM of JUA, JUB, EPA, BTL, and FRS for 15 min in the presence and absence of 500 ng/mL of α-MSH. Intracellular cAMP levels were quantified using a Colorimetric ELISA Kit (Cell Biolabs Inc., San Diego, CA, USA) according to the manufacturer’s instructions. The amount of cAMP was calculated based on the standard curve.

### 4.9. Maintenance of Zebrafish Embryo and Larvae

Inbred AB strains of zebrafish were supplied from the Nakdonggang National Institute of Biological Resources (Sangju, Gyeongsangbuk-do, Republic of Korea). The experiment using zebrafish larvae was approved by the Animal Care and Use Committee of Jeju National University (Jeju, Jeju Special Self-governing Province, Republic of Korea; approval No.: 2021-0030). All methods were carried out in accordance with the approved guidelines [39]. Embryos were obtained using natural mating and maintained in E3 embryo media (34.8 g of NaCl, 1.6 g of KCl, 5.8 g of CaCl_2_·2H_2_O, and 9.78 g of MgCl_2_·6H_2_O in 1 L of distilled water, pH 7.2) supplemented with 1% methylene blue for 48 h and treated with 200 nM of PTU containing E3 media for another 24 h.

### 4.10. Anti-Melanogenic Effect in Zebrafish Larvae

Zebrafish larvae at 3 dpf (*n* = 20) were treated with flavonoid glycosides (each 20 μM) in the presence or absence of 1 µg/mL of α-MSH for 72 h. Images were captured using a stereomicroscope (Olympus, Tokyo, Japan). In a parallel experiment, the heart rate (heartbeats/min) was measured to evaluate the cardiotoxicity of flavonoid glycosides.

### 4.11. Statistical Analysis

The images for RT-PCR and Western blotting were visualized by ImageQuant LAS 500 and transported into Adobe Photoshop (version 8.0). All images were quantified by Image J software (National Institute of Health, Bethesda, VA, USA, www.imagej.net (accessed on 2 June 2021)) and then statistically analyzed by SigmaPlot version 12.0 (Systat Software, San Jose, CA, USA). All data are presented as the mean ± standard error median (SEM). Significant differences between groups were determined using an unpaired one-way ANOVA test with Bonferroni correction. Statistical significance was indicated.

## 5. Conclusions

In this study, we investigated whether five bioactive flavonoid glycosides isolated from the dried mature seeds of *Z. jujuba* var. *inermis* (Bunge) Rehder possessed anti-melanogenic properties. JUB, EPA, and FRS exhibited potent anti-melanogenic effects by inhibiting tyrosinase enzyme activity and the cAMP-CREB-mediated MITF axis. Therefore, apart from other health benefits such as the sedative and hypnotic effects of *Z. jujuba* var. *inermis* (Bunge) Rehder, the anti-melanogenic effect is also profound, which will help in the development of new commercial anti-melanogenic agents.

## Figures and Tables

**Table 1:** Primers and PCR conditions.

Gene *	Primer Sequence (5′–3′)	Size	T_m_	Cycle No.
*MITF*	F: 5′- CCCGTCTCTGGAAACTTGATCG -3′	403 bp	60 °C	27
	R: 5′- CTGTACTCTGAGCAGCAGGTC -3′			
*Tyrosinase*	F: 5′- GTCGTCACCCTGAAAATCCTAACT -3′	110 bp	60 °C	27
	R: 5′- CATCGCATAAAACCTGATGGC -3′			
*GAPDH*	F: 5′- ACCACAGTCCATGCCATCAC -3′	480 bp	60 °C	25
	R: 5′- CACCACCCTGTTGCTGTAGC -3′			

Bp; base pair, T_m_; melting temperature. * MITF; microphthalmia-associated transcription factor, GAPDH; glyceraldehyde 3-phosphate dehydrogenase.

## Data Availability

All data used and analyzed during the current study are available from the corresponding author on reasonable request.
