# Correction: Molagoda et al. Flavonoid Glycosides from *Ziziphus jujuba* var. *inermis* (Bunge) Rehder Seeds Inhibit α-Melanocyte-Stimulating Hormone-Mediated Melanogenesis. *Int. J. Mol. Sci.* 2021, *22*, 7701

**DOI:** 10.3390/ijms27125417

**Published:** 2026-06-16

**Authors:** Ilandarage Menu Neelaka Molagoda, Kyoung-Tae Lee, Athapaththu Mudiyanselage Gihan Kavinda Athapaththu, Yung-Hyun Choi, Jaeyoung Hwang, Su-Jin Sim, Sanghyuck Kang, Gi-Young Kim

**Affiliations:** 1 Department of Marine Life Science, Jeju National University, Jeju 63243, Republic of Korea; neelakagm2012@gmail.com (I.M.N.M.); gihankavinda@yahoo.com (A.M.G.K.A.); 2 Research Institute for Basic Sciences, Jeju National University, Jeju 63243, Republic of Korea; 3 Forest Biomaterials Research Center, National Institute of Forest Science, Jinju 52817, Republic of Korea; leekt99@korea.kr (K.-T.L.); sujin0606@korea.kr (S.-J.S.); 4 Department of Biochemistry, College of Oriental Medicine, Dong-Eui University, Busan 47227, Republic of Korea; choiyh@deu.ac.kr; 5 Department of Chemistry, Gyeongsang National University, Jinju 52828, Republic of Korea; jaeyoung@gnu.ac.kr; 6 Korea Beauty Industry Development Institute, Jeju 63309, Republic of Korea; ksanggh@kbidi.or.kr

In the original publication [1], there was a mistake in Figures 2A and 3D published. Same figures were attached. The corrected Figure 2A and Figure 3D appear below. The authors state that the scientific conclusions are unaffected. This correction was approved by the Academic Editor. The original publication has also been updated.

## Figures and Tables

**Figure 2:**
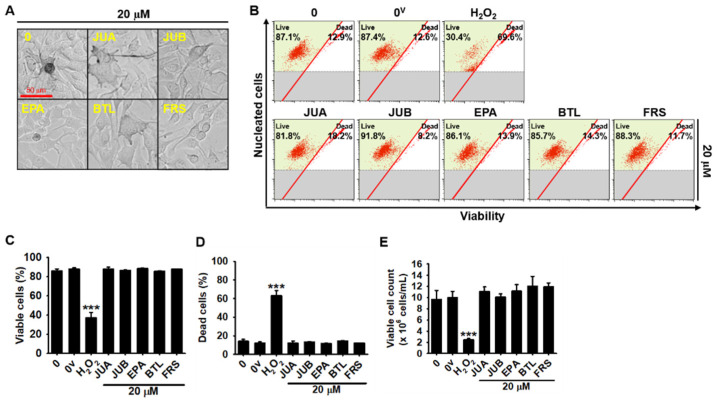
Flavonoid glycosides from *Z. jujuba* var. *inermis* (Bunge) Rehder seeds present no cytotoxicity in B16F10 melanoma cells. B16F10 melanoma cells (5 × 10^4^ cells/mL) were treated with 20 µM of jujuboside A (JUA), jujuboside B (JUB), epiceanothic acid (EPA), betulin (BTL), and 6′′′-feruloylspinosin (FRS) for 96 h. (**A**) Images of the cells were captured using a phase contrast microscope (×20). Scale bar = 50 µm. (**B**) Cell viability was analyzed by a Muse Cell Count and Viability Assay Kit. (**C**) Viable cell (%) and (**D**) dead cell (%) population, and (**E**) viable cell count (×10^6^ cells/mL) are shown. Hydrogen peroxide (H_2_O_2_, 100 µM) was used as a cellular death-inducing positive control. All data are presented as a standard error of the median from three independent experiments (*** *p* < 0.001 vs. untreated cells). 0, untreatment; 0^v^, vehicle control (0.01% DMSO).

**Figure 3:**
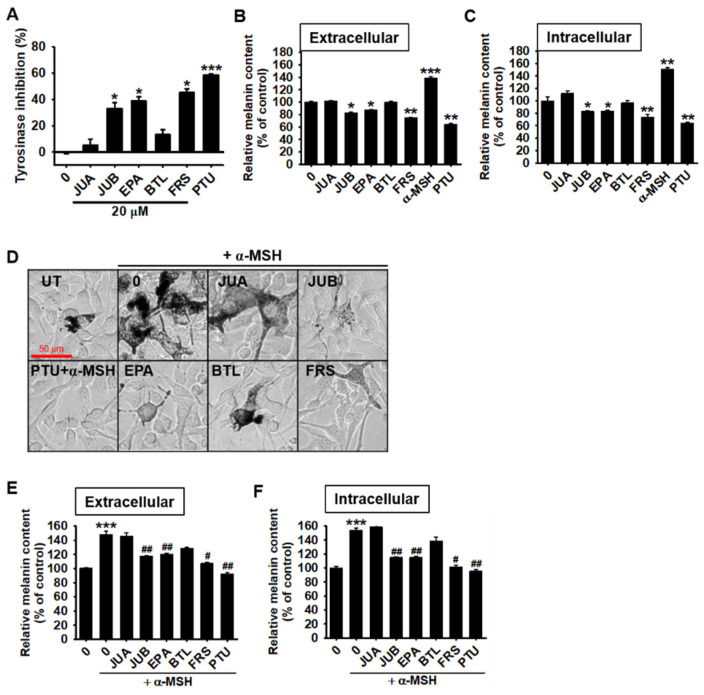
JUB, BTL, and FRS from *Z. jujuba* var. *inermis* (Bunge) Rehder seeds inhibit in vitro tyrosinase activity and melanin production in B16F10 melanoma cells. (**A**) In vitro mushroom tyrosinase enzyme activity in the presence of 20 µM of jujuboside A (JUA), jujuboside B (JUB), epiceanothic acid (EPA), betulin (BTL), and 6′′′-feruloylspinosin (FRS) was determined by oxidation of L-tyrosinase as a substrate. Phenylthiourea (PTU, 200 nM) was used as a positive control. B16F10 murine melanoma cells (5 × 10^4^ cells/mL) were treated with 20 µM of JUA, JUB, EPA, BTL, and FRS for 96 h in the (**B**,**C**) absence and (**D**–**F**) presence of 500 ng/mL of α-melanocyte-stimulating hormone (α-MSH). Intracellular and extracellular melanin was quantified. PTU was used as a negative control. (**D**) Images of the cells were taken with a phase contrast microscope (×20). Scale bar = 50 µm. All data are presented as standard errors of the median from three independent experiments (*** *p* < 0.001, ** *p* < 0.01, and * *p* < 0.05 vs. untreated cells; ^##^ *p* < 0.01 and ^#^ *p* < 0.05 vs. α-MSH-treated cells).
